# Avocado (*Persea americana*) peel: a promising source of bioactive compounds

**DOI:** 10.3389/fnut.2025.1642969

**Published:** 2025-10-01

**Authors:** Morenikeji Abel Oke, Elijah Adegoke Adebayo, Nathaniel Aanu Ajisope, Busayo Mutiat Olowe, Oluwabunmi Susan Fasuan

**Affiliations:** ^1^Department of Pure and Applied Biology, Ladoke Akintola University, Ogbomoso, Nigeria; ^2^Microbiology and Nanobiotechnology Laboratory, Ladoke Akintola University of Technology, Ogbomoso, Nigeria; ^3^Department of Biological Sciences, Bamidele Olumilua University of Education, Science and Technology, Ikere, Nigeria; ^4^Department of Biological Science, Ajayi Crowther University, Oyo, Nigeria

**Keywords:** avocado peel, catechin, procyanidins, chlorogenic acid, maceration, antioxidant

## Abstract

In recent times, the industrial high demand for *Persea americana* (PA) fruits has swiftly increased its production globally. This has resulted in the excessive presence of avocado peel (AVDP) waste as an environmental pollutant since the peel is commonly discarded without any further application. AVDP has been revealed as a key and rich source of manifold nutritional and bioactive components. These include polyphenols, flavonoids, organic acids, hydroxybenzoic, hydroxycinnamic acid derivatives, (epi) catechin derivatives, pro-anthocyanidins, procyanidins, quercetin derivatives. AVDP possessed enriched nutritional profiles ranging from protein, carbohydrate, lipids, fibers, and ashes, with various applications in the medicinal, cosmetics, and food industry. Bioactive components in the AVDP have been linked with several pharmacological properties, like antioxidants, anti-inflammatory, and antimicrobial. The enriched nutritional profile has confirmed AVDP utilization in the food industry as a functional food, food additives, feed formulations, and preservatives. Hence, the valorization of this AVDP recycling to produce diverse materials with potential industrial and medicinal impact is necessary. This review will focus on the nutritional profile and bioactive components of the AVDP, its pharmacological and food industrial applications.

## Introduction

One of the best ways to maintain a healthier and safer environment is by effectively recycling various types of waste within the ecosystem. In addition, recycling environmental waste can significantly contribute to global economic development. Globally, the massive production of agricultural waste products from the agro-industrial sector has recently become a major international issue. Pollutants and waste, especially agricultural by-products, are continuously released into the ecosystem every second, causing numerous problems for the environment. A global waste production of about 2.1 billion tons was reportedly generated in 2016, which is projected to reach 2.59 billion tons by 2030, and predicted to reach 3.4 billion tons by 2050, with agricultural waste constituting a substantial fraction of this total (1–5). Hence, the conversion of these waste products into an effective and useful product or agent, with efficient functional properties, has become one of the attractive areas of research recently. These will efficiently sustain the environmental health by removing them from the environment and transforming them into a valuable industrial product that would potentially enhance or improve the health status of the individuals and the economic status of the nation.

Avocado (AVD) fruit is one of the agricultural products commonly available in the tropics and sub-tropics, widely recognized as a food source for over 8,000 years (6). Globally, the production of AVD fruit is estimated to be around 4 million metric tonnes per year, while Ethiopia is reported to account for about 25,633 metric tonnes annually (7). The industrial processing of fruit juice and oil, along with individual demand for AVDs as an agro-based product, has rapidly increased their production. This surge has led to the generation of substantial quantities of byproducts, including peels, seeds, pulp, and other organic matter, which account for about 30–45% of the total fruit weight (8–13). Wong et al. (14) reported a global production increase of about 52%, which is accompanied by their high nutritive value. So, surprisingly, an upward trend in avocado production from 6,842,058 tons, to 8,978,275 tons was revealed from 2018 to 2022 (15). These byproducts are primarily discarded into the environment. While industries often focus on the immediate treatment of waste, this is usually prompted by uncontrolled decay, contributing to global warming and raising health risks as significant concerns (16–21). This situation poses severe economic and environmental consequences, resulting in estimated annual losses of around 940 billion dollars (22). Notably, AVDP has emerged as the largest part of the AVD consistently released into the environment without any further utilization (9, 13).

However, several studies have investigated and established the diverse functional components composition present in different parts of the AVD, encompassing the peel, pulp, and seed. This composition could be utilized in various industries, such as food, cosmeceuticals, and pharmaceuticals (12, 23–27). Functional foods are food categories with inherent health-promoting functional components that proffer advantages beyond basic nutrition, but incorporate the potential improvement of overall health and reducing the risk of disease. AVDPs are shown to be a rich source of valuable nutritional components, which are key parts of food and diet, while their diverse bioactive compounds contribute to potential treatment options for many diseases (25, 26). Valorizing this waste could be achieved by converting it into a value-added product through the extraction of important constituents like protein and phytochemicals that can be utilized in various industries in diverse ways (12, 25, 26).

The emancipation of nanobiotechnology, which incorporates the application and adoption of biological materials to synthesize different nanoparticles, have received great attention with great importance of the synthesized nanoparticles in diverse fields of life (28–31). The application of biological materials has been linked to their eco-friendliness, low cost, ease of availability, and diverse bioactive composition. Biological materials, via microorganism and their metabolites, plant materials, including agro-industrial waste, have been used for the synthesis of nanoparticles (28–30, 32, 33). AVDPs have been repeatedly used in synthesizing different nanoparticles, including silver nanoparticles, gold nanoparticles, zinc oxide nanoparticles, and many more (28, 33). These synthesized nanoparticles mediated by AVDP have been shown with different biological activities better than the extract alone (28). Hence, this review will focus on the nutritional profile and bioactive constituents of the AVDP, its pharmacological and food industrial applications.

### Taxonomy of *Persea americana*

**Figure 1:**
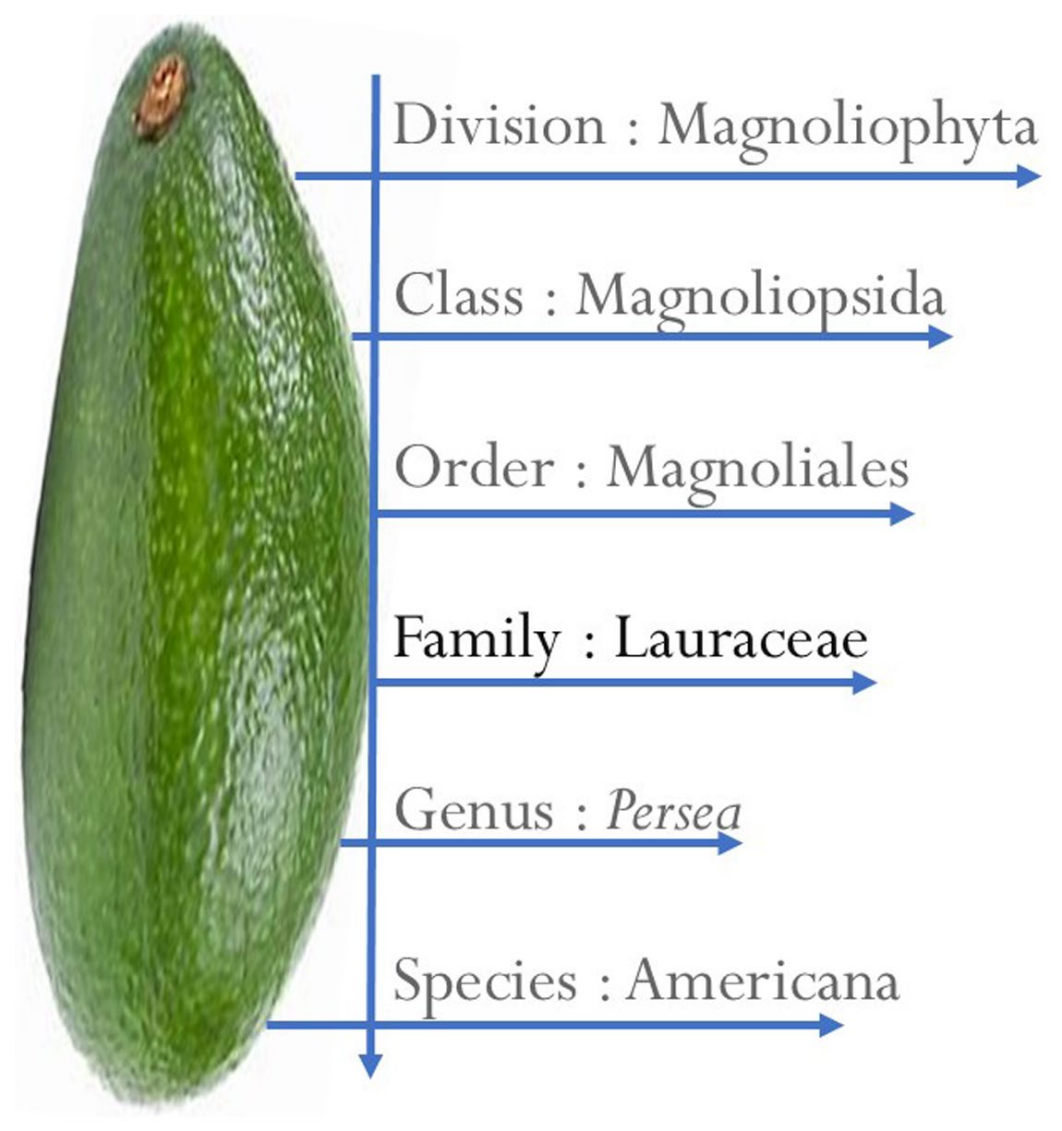
Taxonomy of avocado.

AVD is a fruit classified under the genus *Persea* and the family Lauraceae, (Figure 1), which includes about 50 species primarily cultivated in warm temperate climates (28). The Lauraceae family consists of dicotyledonous perennial plants that are native to Mexico. The earliest record of AVD cultivation dates back to Mexico as early as 500 BC, but it is now grown in various tropical and subtropical regions worldwide (25, 34, 186). The term AVD originates from the Aztecs and is derived from Nahuacatl. However, this fruit is known by various names in different countries, including aguacate, cupandra, avocatier, cura, abacate, alligator pear, butter pear, and palta (23, 25, 35).

AVD is botanically classified into three groups based on their origin, cultivation conditions, and other features of the fruit: the Mexican (PA var. drymifolia), Guatemalan (*Persea nubigena* var*. guatemalensis*), and West Indian (PA var. americana) (23, 36). AVDs come in several different varieties, among which the cultivars, Hass and Fuerte, remain the most commonly cultivated (25, 36, 37). More than 500 varieties of AVD have been identified, including Hass, Lamb Hass, Shepard, Reed, Wurtz, Fuerte, Sharwil, Zutano, Ettinger, and Edranol, among others (23, 38–40). However, various issues, such as the cultivation period, protein and fat composition, their ability to withstand adverse environmental challenges, and postharvest damage, have reduced most of them from commercial production.

AVDs differ greatly based on their weight, size, form, and flavor, although the most renowned difference is the color of the ripened skin/peel (23, 41). Hass AVD (PA) is reported as the most cultivated and consumed AVD cultivar globally. It remains the most widely studied, with approximately 95 percent of the total commercialized capacity generated by the Guatemalan/Mexican hybrid in the United States (24, 42, 43).

### Morphological appearance

Generally, AVD (*P. americana*) is of diverse varieties, all of which were broadly classified into three major categories. Each variety was named based on its geographical location, where it originated or was domesticated (10). In the West Indian, it is scientifically known as *P. americana*, in Guatemala referred to as *P. guatemalensis*, while it’s known as *P. drymifolia* by the Mexicans. In addition, AVDs are furnished with dissimilar morphological characteristics based on the texture and color of the peel, coupled with the fruit size at large. For instance, a higher quantity of oil content with a small size was associated with the Mexican variety of AVDs, which is higher than that of the West Indian variety (10).

AVD trees are about 20 m tall and are tropical evergreens. The tree has a thick bark with a grayish-brown color and broad leaves between 7 and 14 cm long. They have flowers ranging between 1 and 1.3 cm in width, with color yellow or green. The fruit itself is a drupe, and each AVD fruit has a big seed. Their cultivar determined their size (10, 44–46). AVD is composed of the edible part (pulp or mesocarp), a hard black cover, and a rough skin. As a whole AVD fruit consist of the outer flesh skin (exocarp), the edible part (mesocarp) coupled with the inner seed (endocarp) weighing about 100 and 1,000 g, and of about 33% of the fruit total weight (23, 25, 45, 47). AVDs were unique in ripening; the ripening process starts after harvesting, which could be up to about 5 to 7 days at ambient temperature, but never ripens on the tree (35).

### Global production and distribution

**Figure 2:**
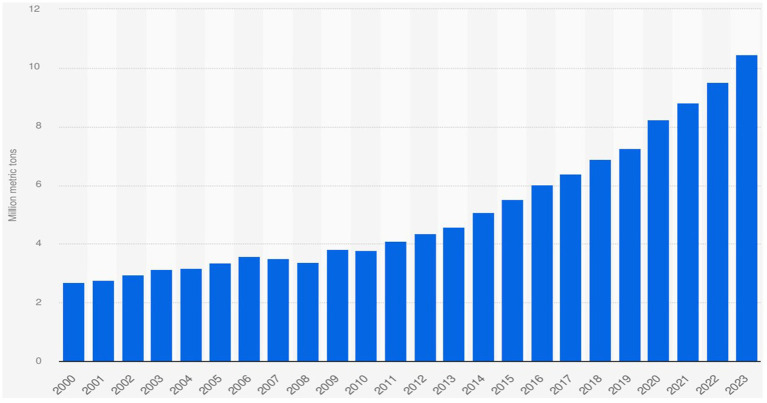
Avocado production volume worldwide from 2000 to 2023 (178).

AVDs are produced in large quantities each year, representing one of the fruits with the highest production and consumption rates worldwide, with significantly increasing demand as illustrated in Figure 2 (8, 11). AVD production rose from 2.2 million tons to 6.4 million tones globally between 1995 and 2018, with approximately 10.47 million metric tons in 2023 (35, 48). AVD production has received a significant increase, doubling its annual production rate over the last 10 years (Emir (49)).

The first cultivation of AVD is believed to have taken place around 500 BC in Mexico, which remains the leading producer, accounting for approximately 30% of annual AVD production globally, about 1.8 million tons per year in 2019, while the production increased to 2.3 million tons in 2020 and 2.5 million tons in 2022 (48, 50). Colombia is the second-largest producer with 1.0 million tons (12%), followed by Peru, the third-largest producer globally, yielding over 866 thousand tons annually (10%) (15, 51). Additionally, the Dominican Republic, Indonesia, Kenya, and Brazil were responsible for approximately 7, 6, 5, and 3% of global AVD production, respectively (10). In 2022, based on the overall productions per continent, America leads, followed by Africa, Asia, Europe, and Oceania with 72, 14, 11, 2 and 1%, respectively.

The consumption rate of AVD was reported to be about 5.8 million tons in 2018, which significantly increased to 7.1 million tons in 2020. The Americas were shown to be the largest AVD consumer with 63% of global consumption in 2020 (44). Asia was the second-largest consumer with 14%, followed by Africa with 11% with the least consumption of 2% in Oceania. A market price of about US$1.70 billion was evaluated in 2018, while a rise to nearly US$2.70 billion is predicted by 2024 (15, 40, 52). The United States takes the lead in AVD consumption, followed by Mexico, Colombia, Indonesia, and the Dominican Republic, with 1.2 million, 1.0 million, 719 thousand, 557 thousand, and 500 thousand tons of consumption rate, respectively, in the year 2020 (15).

The AVD pulp is primarily utilized in industrial processes for various purposes, including oil extraction from the pulp and the production of paste, among other products. In contrast, the other parts (peels and seeds) are discarded into the environment, resulting in approximately 2.42 million tons of by-products (40). The AVDP accounts for about 13–18% of the total weight of the fresh fruit (23, 53–55).

### Avocado peels functional components

#### Nutritional profile

**Figure 3:**
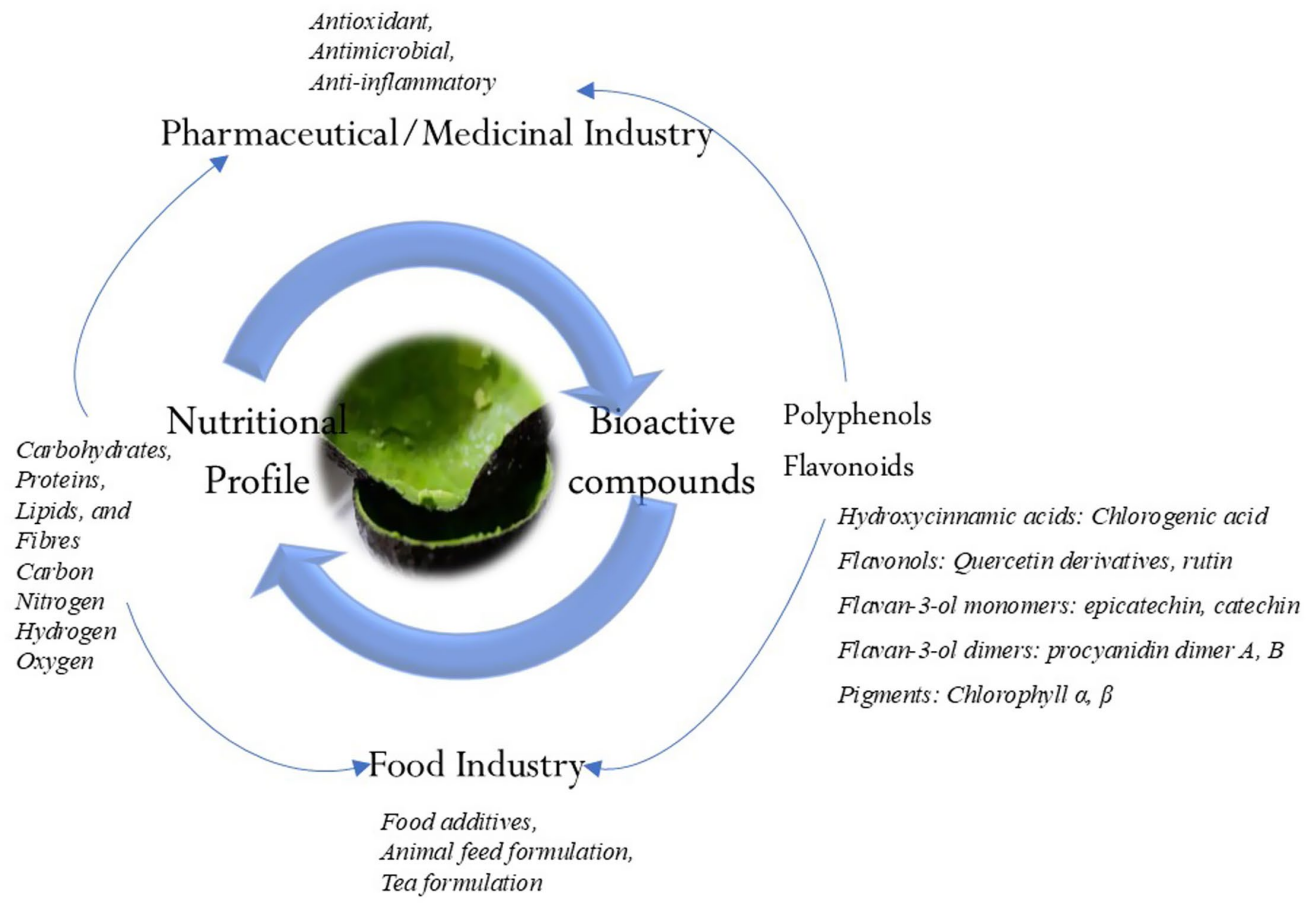
Nutritional and phytochemical constituents of AVDP.

AVDP has been established by several studies as a significant source of valuable nutritional constituents (Table 1). It has been shown to contain carbohydrates, proteins, lipids, and fibers at levels of 62–73.3%, 4–8.3%, 4.4–9.1, and 50%, respectively (6, 56). Additionally, the elements within the AVDP include carbon (49.83%), nitrogen (0.97%), hydrogen (5.71%), and oxygen (42.2%) (35, 57). Figure 3 illustrates some nutritional and phytochemical constituents of AVDP. Hence, AVDPs remain a promising material as functional foods in the food industry and for producing nutraceuticals, cosmeceuticals, and pharmaceutical products. They can also serve as a natural source for producing eco-friendly adsorbents (35, 58–60).

**Table 1:** Extraction methods, bioactive components, and biological properties of some reported AVDP.

Cultivars	Region	Methods	Solvent	Bioactive components	Biological properties	Concentrations	Experiment	Ref
NS	Nigeria	Maceration. % Recovery (14–21%)	Acetones, water, ethanol, methanol, and water at a concentration of 90% (V/V)	Phenol, flavonoids	Antioxidant and Antibacterial Potentials	5,10,15,20,25,100,150,200,250,300,350,400,450, and 500 mg/mL	*In vitro*	Bassey et al. (179)
NS	India	Maceration % Recovery (14–21%)	NS	Alkaloids, flavonoids, phenols, tannins, glycosides	Antioxidant and Antibacterial Potentials	100,150, 250,350,450 mg/mL	*In vitro*	Kamaraj et al. (113)
Hass	Chile	Microwave-assisted Hydrolysis. % recovery (18.56–42.58%) eight-fold higher phenols than conventional solid–liquid extraction	Ethanol, Water, the organic fraction, the aqueous fraction, and the acid-microwave hydrolyzed APE	Higher phenolic composition, including proanthocyanidin	Antioxidant activity	20 μL	*In vitro*	Trujillo-Mayol et al. (132)
Hass	Portugal	Maceration % Recovery (14–21%)	Hydroethanolic extracts (ethanol: water, 80:20 v/v)	(Epi)catechin derivatives, chlorogenic derivatives	Antioxidant, antimicrobial, and cytotoxic activities	250 μL	*In vitro*	Melgar et al. (34)
Hass and Fuerte		Maceration % Recovery (14–21%)	-	Polyphenols	Anticancer, antidiabetic, and antihypertensive effects		*In vitro*	Araújo et al. (23)
Hass and Fuerte	Spain	Maceration % Recovery (14–21%)	Ethyl acetate, 70% acetone, and 70% methanol extracts acetone/water (70:30 v/v); or methanol/water (70:30 v/v)	Catechins, procyanidins, and hydroxycinnamic acids	Antioxidant and Antibacterial Potentials	200 μL	*In vitro*	Rodríguez-Carpena et al. (54)
Haas	Brazil	Maceration % Recovery (14–21%)	Dried peels used in a functional beverage formulation (tea rich in antioxidants)	Phenolic compounds (10,848.27 ± 162.34 mg GAE kg-1), flavonoids (1,360.34 ± 188.65 mg EQ kg-1).	Antioxidant activity, Microbiological assay, and sensory analysis	250 μL	*In vivo and in vitro*	Rotta et al. (68)
Hass	Spain	Maceration % Recovery (14–21%)	Ethanol-water mixtures	Phenolic acids (hydroxybenzoic and hydroxycinnamic acids), flavonoids (flavanols, flavanonols, flavones, flavanones and chalcone, phenylethanoids and lignans)	Antioxidant, Anticancer (using Caco-2, A549, and HeLa cell lines) (using Caco-2, A549, and HeLa cell lines)	8, 16, 32, 63, 125, 250, 500, and 1,000 μg/mL	*In vitro*	Rodríguez-Martínez et al. (11)
NS	Indonesia	Maceration % Recovery (14–21%)	Methanol	Phenol, flavonoids, tannin, saponin, and alkaloid	Antioxidant	20, 40, 60, 80, 100 ppm	*In vitro*	Rahman et al. (119)
Hass	Brazil	Maceration % Recovery (14–21%)		Vanillic acid, ferulic acid, gallic acid, hesperidin, procyanidins, dimers, and trimers in various shapes	Antioxidant	200 μL	*In vitro*	Santana et al. (180)
Hass and Fuerte	Brazil	Maceration sonicated in an ultrasonic bath Unique. % Recovery (20–46%)	Ethanol/water, 80/20 v/v	Catechin, epicatechin, procyanidins B 1 and B 2, and trans-5-O-caffeoyl-D-quinic acid	Antioxidant, anti-inflammatory, and cytotoxic properties	0.1, 0.5, 1, 5, 10, 50, and 100 μg/mL	*In vitro*	Tremocoldi et al. (69)
Hass	Spain	Accelerated solvent extraction % Recovery (90–95%)	Water and ethanol	Sixty-one compounds belonging to eleven families were identified. Procyanidins, flavonols, hydroxybenzoic, and hydroxycinnamic acids were the most common compounds	Antioxidant and Antibacterial	250 μL	*In vitro*	Figueroa et al. (92)
NS	Colombia	Maceration % Recovery (14–21%)	70% aqueous acetone and 80% methanol	Organic acids, hydroxycinnamic acids, catechins, glycosylated flavonoids, dimeric and trimeric procyanidins, epicatechin, six quercetin derivatives, four dimeric procyanidins (three type B and one type A), three trimeric procyanidins (two type B and one type A)	Antioxidant properties	100 μL	*In vitro*	Rosero et al. (71)
NS	Portugal	Maceration % Recovery (14–21%)	Ethanolic	Phenolic compounds	Antioxidant and antibacterial properties	250 μL	*In vitro*	Ferreira et al. (90)
Hass	Spain	Maceration % Recovery (14–21%)	Optimal consumption ripeness (CRA), whereas half of the avocados were left until overripeness (ORA).	34 recoveries, while 5 came from AVDP, including quinic acid, chlorogenic acid, quercetin-3,4′-diglucoside, quercetin-3-O-arabinosyl-glucoside, and rutin	NS	NS	*In vitro*	López-Cobo et al. (64)
Hass and Shepard	Australia	Maceration % Recovery (14–21%)	Methanol (80%) extraction with solid to solvent ratio 1:8 in a shaking water bath at 60 °C	Four (4) polyphenolic classes: flavanol monomers, proanthocyanidins, hydroxycinnamic acids, and flavonol glycosides	Antioxidant properties	250 μL	*In vitro*	Kosińska et al. (59)
Fortuna	Brazil	Maceration % Recovery (14–21%)	Hexane and ethanol	Fifty-five metabolites were detected in the extracts, consisting mainly of phenolic acids, flavonoids, and alkaloids	Antioxidant capacity, acetylcholinesterase inhibition, and neuroprotective capacity	500 μg/mL, 10 mg/mL	*In vivo and in vitro*	da Silva et al. (181)
NS	Nigeria	Methanol % Recovery (14–21%)	n-hexane extract, when administered	NS	Antihypertensive activity	at a dose of 100, 200, and 400 mg/kg	*In vivo*	Mamza et al. (182)
NS	Saudi Arabia	Soxhlet extraction and cold maceration % Recovery 14 to 29.93%	Methanolic extract	Phenolic acids, flavonoids, and alkaloids	Antiparasitic	100, 50 and 25 mg/L	*In vivo*	Al-Otaibi et al. (183)
Hass	Chile	Maceration % Recovery (14–21%)	Water	NS	Alterations of Colonic Homeostasis	1800 and 2000 kcal/day for women and men, 163 respectively; 15% proteins, 58% carbohydrates, and 27% fat	*In vivo*	Cires et al. (184)
Pinkerton	Greece	Maceration % Recovery (14–21%)	Water	Phenolic acids, flavonoids, and alkaloids	Antioxidant capacity, antimicrobial properties	50 μL, 25 μL	*In vivo and in vitro*	Skenderidis et al. (129)
NS	Nigeria	Maceration % Recovery (14–21%)	Hydroethanolic extract	Determine its anxiolytic, learning, and memory potential	Starvation and re-feeding on brain oxidative stress markers	50 mg/kg body weight	*In vivo*	Ilochi and Chuemere (185)

The percentage recovery of each extraction method varied based on several factors, including the curvatures, extraction solvents, and extraction conditions like Temperature and others. NS, Not stated. %, Percentage.

Nyong (61) examined the nutritional components of flour made from fifteen (15) AVD seeds and peels collected at a market in River State, Calabar, Nigeria. The results showed that AVDP flour contained approximately 13.00% moisture, 12.00% ash, 15.80% lipids, 13.00% fiber, 25.46% protein, and 20.74% carbohydrates. The moisture, ash, and protein contents of AVDP flour were similar to those of AVD seed flour, while the fiber and carbohydrate contents were higher in AVDP flour compared to seed flour. These findings indicate that AVDPs and seeds have substantial nutrient profiles capable of meeting the body’s protein and fat requirements. Therefore, they could be used as potential functional foods in formulations for both humans and animals.

Teshome et al. (62) reported that AVDPs are rich in proximate composition, including moisture, ash, protein, fat, carbohydrate, and fiber. They observed a higher carbohydrate content compared to other by-product fruits such as apple pomace, ripe mango peel flour, banana peel, AVD seed, raw pineapple peel, raw papaya peel, raw papaya seed, grape pomace, and citrus peel. Additionally, the mineral composition (mg/100 g) of AVDP includes potassium (K), calcium (Ca), sodium (Na), magnesium (Mg), iron (Fe), zinc (Zn), copper (Cu), and manganese (Mn), with respective values of 899.8, 679.3, 21.1, 46.9, 2.3, 1.6, 14.5, and 1.4 mg/100 g. High levels of K and low Na amounts are considered beneficial for individuals on low-sodium diets and can help protect against heart-related diseases. All these factors support its potential use as a functional food ingredient in the food industry.

The AVDP were categorized into raw, oven-dried, and freeze-dried groups, and each was analyzed for nutritional and mineral compositions. The raw peel, oven-dried peel, and freeze-dried peel contain 65.7, 4.0, and 2.3% moisture on a wet basis (%WB); 1.5, 2.0, and 1.7 grams of ash; 6.3, 6.4, and 6.7 grams of protein; 3.5, 4.7, and 2.4 grams of lipid; and 46.9, 43.9, and 43.5 grams of fiber, expressed as a percentage of the fruit part on a dried basis (g per 100 g DB), respectively. Significantly higher values of moisture (65.7%) and fiber (46.9 g) were observed in raw peel, while ash content (1.7 g) and protein content (6.7 g) were higher in the freeze-dried peel, along with lipid (4.7 g). Additionally, the mineral composition of the oven-dried sample showed contents of K (899.0 mg), Ca (679.3 mg), Na (21.1 mg), Mg (46.9 mg), Fe (2.3 mg), Zn (1.6 mg), Cu (14.5 mg), and Mn (1.4 mg) per 100 g of the dried fruit part (63).

#### Bioactive profile

**Figure 4:**
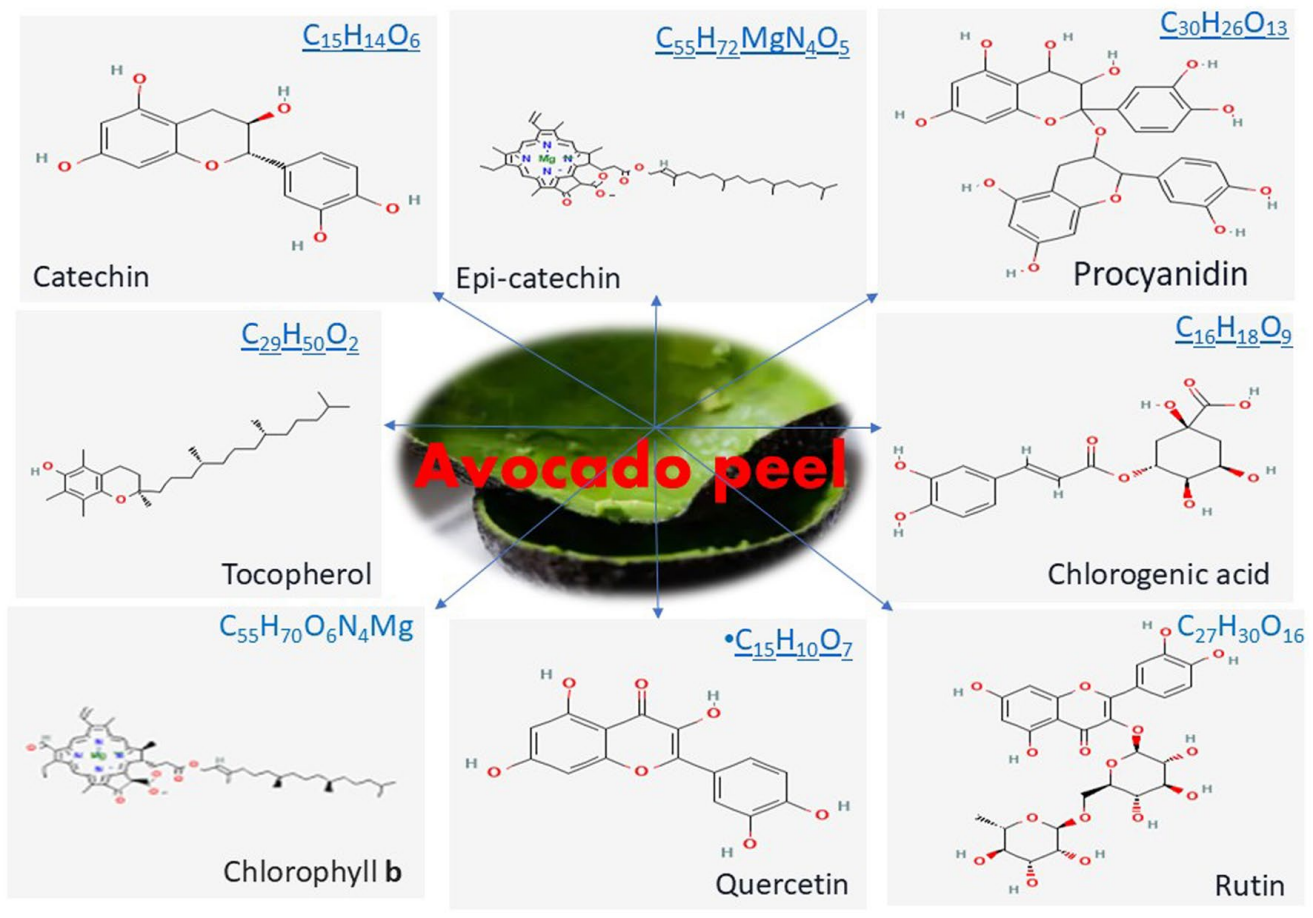
Chemical structure of common phenolic and flavonoid compounds in AVDP.

AVDP is rich and serves as a valuable source of bioactive components as shown in Figure 3 (53, 64). Studies have established the presence of various bioactive components, including organic acids, phenolic acids, and phenolic alcoholic derivatives (Table 1). They also identify flavonoids, quercetin and its derivatives, catechins, procyanidins, chlorophyll a and b, chlorogenic acid, and quercetin. Additionally, some studies have reported 1,2-dihydroxybenzene, 2,3-dihydroxybenzoic acid, gallic acid, rutin trihydrate, syringic acid, and caffeic acid as part of the phenolic constituents found in AVDP (65–67). Figure 4 displays the chemical structure of some common phenolic and flavonoid compounds in AVDP.

AVDPs contain significantly higher phenolic content and antioxidant activity compared to the edible part of the AVD fruit. However, a fresh AVDP was reported to contain phenolic content ranging from 0.6 to 6.8 mg GAE/g sample, while dry AVDP had been shown with a range from 4.3 to 120.3 mg GAE/g sample for dry AVDP, which could vary based on the cultivars (34, 35, 68, 69). Additionally, dry AVDPs have superior total phenolic content (TPC) and antioxidant activity compared to other tropical fruit peels, including banana, melon, passion fruit, papaya, pineapple, and watermelon, while fresh peels exhibit the highest flavonoid content (35, 55, 64, 70).

More than thirty (30) individual phenolic compounds, along with higher polymeric compounds, have been identified in AVDPs. They are categorized into three (3) groups: hydroxycinnamic acids, flavanols, and flavan-3-ols (35). Chlorogenic acid (5-O-caffeoylquinic acid) is recognized as the main hydroxycinnamic acid in AVDP, while quercetin derivatives are noted as the dominant flavanols in AVDPs (34, 35, 59, 64). Flavan-3-ols were reported to be highest in AVDPs (34). Epicatechin, catechin, and A- and B-type dimers are recognized as subgroups of procyanidins, the main polyphenols (55). The level of procyanidin in AVDP has been reported to be comparable to that in natural cocoa powder, which is known for its exceptional procyanidin content. Rosero et al. (71) explore the phenolic composition of AVDPs and Avocado seeds (AVDS). The findings reveal several bioactive constituents, particularly the phenolic compounds, including catechins, procyanidins, and others, identified in the most active fractions of AVD by-products. The fractions with the highest antioxidant activity contained phenolic compounds of higher molecular weight (condensed tannins).

### Factors influencing the kind and quantity of functional components recovery from AVDP

#### Morphological appearance

The quantity and quality of chemical compounds in AVDP differ based on factors such as the ripening and maturation level, the conditions under which the AVD is grown, the AVD variety, the region or country of origin, and the geographical locations of AVD plant growth (25, 35, 59). Wang et al. (55) present Hass variety among eight different cultivars examined, which has the highest phenolic content (51.6 mg GAE/g) and ranks as the third highest antioxidant activity by the ORAC assay (428.8 μmol TE/g) in the peels compared to other varieties.

The phenolic content and antioxidant potential of AVDPs from two different cultivars, Hass and Shepard, were evaluated by Kosińska et al. (59). In this study, Hass cultivar peel has catechin and procyanidin dimers, but absent in the Shepard cultivar peels, while caffeoylquinic acid and quercetin derivatives were present in both varieties. Tremocoldi et al. (69) reported a higher TPC in the Fuerte variety peels (120.3 mg GAE/g of dry AVD) compared to the Hass variety (63.5 mg GAE/g of dry AVD). During and after the maturation stage, AVDP texture and color are altered, which affects the types and amounts of bioactive compounds along with their biological activities (36, 72). The Persin content of Hass AVDPs was monitored throughout the maturation stage and was found to decrease, with 30% of overripe peel containing less total Persin. The concentrations were also observed to decline with an increasing number of ripening and storage; total epicatechin content reduced between the early and late harvest seasons. In contrast, the differences observed in storage and ripening were much less pronounced than the changes related to maturation (72).

#### Extraction technique

**Figure 5:**
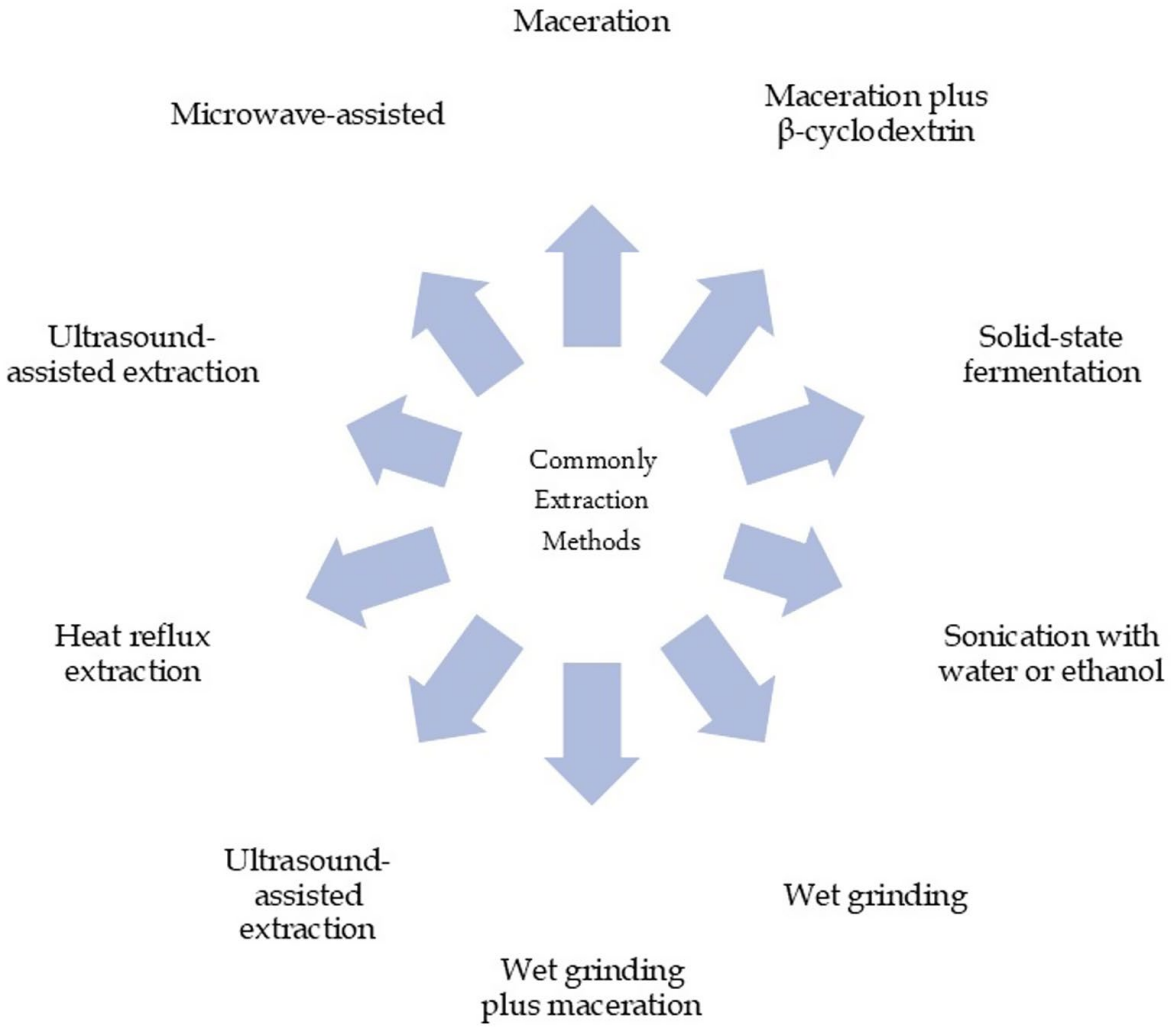
Commonly reported extraction techniques for AVDP.

The extraction techniques and conditions under which the extractions occur significantly impact the quality and quantity of the recovered chemical compounds, as well as their biological activities. Recovery of functional components, including phenolic contents, from the AVDPs was performed using both conventional and non-conventional extraction methods (Table 1). Generally, conventional extraction methods, such as maceration (M) or maceration assisted by ultrasonic bath, hydro-distillation, soxhlet, and hydrodistillation, have commonly been utilized and are still preferred in the industry due to their ease of handling, compatibility with ambient conditions, and relatively mild temperatures (26, 49, 53, 64, 70, 73–77). Figure 5 presents some commonly used extraction techniques for AVDP.

Several other non-conventional methods of extractions for the efficient recovery of chemical compounds from the AVDPs have been studied and reported. Some of these approaches includes heat reflux extraction (78); ultrasound-assisted extraction (UAE) (79, 80); microwave-assisted extraction (81), enzyme-based extraction (EBE), surfactant-mediated extraction, pulsed-electric field extraction, centrifugal partition extraction, pressurized liquid extraction, supercritical fluid extraction, three-phase partitioning, high voltage electric discharge plasma, natural deep eutectic solvents extraction, and two-phase aqueous systems, among others are effective and come with some advantages, including improve efficiency thereby reducing environmental pollution and other associated risks (82–86). However, some utilize organic solvents such as methanol, ethanol, chloroform, acetone, etc., which can have negative effects on the ecosystem, causing great damage both to humans and to the environment, in addition to their high cost (85, 87–89). Besides, the non-conventional techniques require specialized equipment.

Applications of conventional technology via M have been studied using different extraction parameters and solvents to depict the specific and total amount of bioactive compounds, coupled with the biomedical evaluations of the extracts. Some of the reported parameters includes methanol at 80%, ethanol at 20, 60, 80 and 95%, acetone at 70 and 80%, acetone/water/acetic acid at 70:29.7:0.3, v/v/v/v, ethyl acetate, acetone/water at 70:30 v/v, methanol/water at 70:30 v/v, methanol with 0.10% trifluoroacetic acid, absolute ethanol and boiled water (26, 34, 52–55, 59, 64, 69, 72, 90–97).

A complete analysis of the bioactive components present in AVDP was evaluated by Figueroa et al. (93) using an accelerated solvent extraction approach, with water and ethanol as extraction solvents. Sixty-one (61) compounds from several structural classes were identified. Out of the five identified hydroxybenzoic acids, only three were reported before using dynamic mercerization extractions approaches (77), while the other two, gentisic acid and benzoic acid, were first reported in this study. Five different groups of hydroxycinnamic acids were identified in this study. Caffeic acid and p-coumaric acid have been reported by Wong et al. (77) and Saavedra et al. (26), respectively, using dynamic maceration approaches, while 3-0-caffeoylquinic acid was reported by Rodríguez-Carpena et al. (54) using Accelerated solvent extraction approaches. However, isomer 4-O-caffeoylquinic acid, hydroxytyrosol glucoside, tyrosol-glucoside, tyrosol-hexoside-pentoside, phenolic alcohol derivatives, quercetin glucuronide, quercetin 3-glucoside, kaempferol O-glucosylrhamnoside, and flavonoid group were reported in AVDP for the first time. Flavanols, catechin, epicatechin, and procyanidins were quantified in larger amounts than those earlier reported due to different extraction approaches (53, 54, 59, 64, 70, 75, 77, 97).

Martínez-Gutiérrez et al. (49) studied the impact of six different methods of extractions, including M plus *β*-cyclodextrin, solid-state fermentation (SSF), sonication with water or ethanol, wet grinding, and wet grinding plus maceration (WGM) in recovering the bioactive components from the AVDP. Twenty-seven (27) phenolic compounds were recovered, while thirty-eight (38) compounds were identified using GC–MS analysis. It was found that the used extraction approach had a great influence on the quantity of the recovered bioactive compounds. The WGM presents the highest total phenols, epicatechin, and chlorogenic acid contents among the six different extraction methods adopted. Thus, wet WGM displayed good yields of phenolics while using an easily accessible and environmentally friendly technology. This is similar to the study carried out by Emir et al. (49), six different extraction methods were utilized to recover phenols, epicatechin, and chlorogenic acid from the AVDPs. The highest recovery was obtained using the WGM method, allowing for an eco-friendly reaction using available technology.

Marović et al. (98) studied the effects of various drying techniques, including hot air, vacuum, and hot-air microwave (HAMD), on the content of fatty acids and tocopherols in AVDPs, Avocado pulp (AVDPP), and AVDS. All methods were subjected to the same temperature (60 °C) but different durations: 35 min, 150 min, and 200 min, respectively. Oleic acid exhibited the highest percentage, followed by palmitic and linoleic acids, with ranges of 41.28–57.93%, 19.90–29.45%, and 8.44–14.95%, respectively. Significant reduction in oleic acid content was observed in the drying samples, while palmitic acid showed the greatest stability. The dried AVDPP and AVDP samples contained higher oleic and linoleic acid concentrations compared to those from the vacuum and HAMD methods. However, samples prepared using HAMD contain a higher *α*-linolenic acid concentration. The findings indicated that HAMD is the most efficient technique. A consistently higher total tocopherol content was noted. Therefore, AVDP is suggested to offer promising health benefits due to its higher content of this valuable chemical component.

The influence of temperature and solvent-feed (S/F) ratio on the extraction yield, as well as the quality and quantity of chemical components, including TPC and total flavonoid content (TFC), and antioxidant capacity of AVDPs extract at two different maturation stages was investigated by García-Ramón et al. (2). Solid–liquid extraction through M was employed to determine the amount of phenols present and their antioxidant activities. Notably, the unripe AVDPs yielded the highest extraction rates, TPC, TFC, and antioxidant capacity. The extraction utilized 40% ethanol at 49.3 °C with a solvent-to-feed ratio of 14.3 mL/g for 60 min. Vanillic acid and 4-hydroxyphenylacetic acid remained the main recovered phenolic components. Therefore, the study suggests that AVDPs serve as a natural source of phenolic compounds, presenting industrial significance, especially in food formulations and functional foods, as an environmentally friendly and health-conscious alternative to synthetic antioxidants.

The efficacy of UAE arises from acoustic cavitation, which enhances mass transfer across cell membranes (99, 100). Conversely, the effectiveness of EBE relies on an enzyme’s capability to degrade the cell wall, resulting in a greater release of intracellular components (101). Hefzalrahman et al. (102) examined the effects of EBE and UAE on the recovery of bioactive constituents from AVDPs. The bioactive composition of the extract was identified, and antioxidant activity was determined. Benzoic acid, vanillic acid, resveratrol, and syringic acid were identified as the main phenolic compounds. Both extraction methods produced extracts with strong antioxidant potential; however, the enzyme-assisted extract demonstrated a higher antioxidant capacity than the ultrasound-assisted extract. This indicates that more phenolic compounds were released or recovered through enzyme-assisted extraction, leading to enhanced antioxidant potential. Therefore, AVDP extract is regarded as a promising antioxidant agent, which is essential in food as a functional food and preservatives, drug formulations, and cosmeceutical industries.

The applications of various drying methods, including oven drying, lyophilization, airflow rate, and loading density when using drying as a pretreatment method, significantly affect the total concentrations of phenolic and flavonoid content in AVDP. Higher drying temperatures and airflow rates result in lower TPC in AVDP (26). Reports indicate that lyophilization treatment decreases phenolic content in AVDP, while the oven drying process increases it. Conversely, the TFC in AVDP has been shown to decrease with both lyophilization and oven drying compared to raw samples. However, under similar drying conditions for phenolic compounds, some were found to increase while others decreased (70). The use of heat as a treatment method could effectively liberate phytochemicals into their free forms, thereby increasing the total of each bioactive compound along with their biological activities. Heat applications may lead to the degradation of thermo-sensitive phenolic and flavonoid compounds, causing the denaturation of these compounds (103).

Typically, the concentration of the solvent, the solvent-to-feed ratio, and temperature are the key parameters that influence extraction efficiency and minimize the loss of these compounds, especially in the solid–liquid extraction method (89, 104–107). The temperature at which plant materials are subjected significantly impacts the phenol content. Polyphenol contents are better preserved at specific temperatures due to the inactivation of the enzyme polyphenolic oxidase. However, at any temperature exceeding this, heat-sensitive polyphenols may be destroyed (66, 67).

### Industrial applications of AVDPs

The large production of AVD worldwide results in the release of a substantial number of peels into the environment. The unexplored bioactive constituents of these peels lead to the loss of several valuable phytochemicals, which could be used in the production of various products of high economic value (6, 108, 109). Many economically valuable, phytochemically rich materials are often lost from the large amount of AVDP generated daily from its processing (108, 109). AVDPs are a rich source of phytochemicals, which can be utilized in providing various nutritional and therapeutic solutions. Different studies have established that AVDPs contain higher phenolic contents than the seeds. These phenolic contents have been linked to various biomedical applications. Traditionally, the AVD seeds and peels are currently used as active materials in producing foods and beverages (40).

AVDPs are rich in bioactive compounds, particularly phenolic content, even more so than the pulp and seed, which are highly beneficial in the food, pharmaceutical, and other industries (59, 68, 69). Recently, there has been increasing interest in investigating the bioactive composition along with their applications in various fields. Numerous biological activities are associated with AVDPs due to presence of beneficial components, combined with the absence of potentially toxic or harmful substances commonly found in many dermatological products, establishes AVDPs as a preferred natural raw material for cosmetics, in addition to their various applications in the food and pharmaceutical sectors (25, 59, 69, 94, 110, 111). AVDP has been recognized as a promising source of essential compounds for food, pharmaceutical, and other industrial uses. It serves as a valuable source of phenolic content, which is greater than that found in the pulp and seed (68, 69).

### Functional potential and industry

The pharmaceutical applications of the AVDP as an antioxidant, anticancer, antibacterial, and insecticidal agent have been reported (112–115). Okoye (116) reported the chemoprotective potential of these polyphenols against cancer activities. Another study noted the platelet aggregation inhibition properties, anti-allergenic, antihypoglycemic (117), anti-inflammatory, and antioxidant properties, as well as the ability to improve lipid metabolism of these polyphenols in the AVDP (118).

#### Antioxidant

Some bioactive compounds in AVDP have significant antioxidant potential. Procyanidins, chlorogenic acid, pigments like chlorophyll, and flavonoids such as flavonols are antioxidant compounds found in AVDP that exhibit free radical scavenging activity, helping to prevent cardiovascular diseases, cancer, and neurodegenerative conditions. While chlorophyll, an antioxidant pigment, is present in AVDP, it is not the dominant antioxidant compound due to the weak correlation between pigment concentration and antioxidant activity (35, 55). Rahman et al. (119) investigated the antioxidant potential of the AVDP extract using methanol as the extraction solvent. Phenols, flavonoids, tannins, saponins, and alkaloids were identified with total values of 21.833 mg/100 g extract, 2.607 mg/100 g extract, 38.357 mg/100 g extract, 8.874 mg, and 9.95 mg CE/g extract, respectively. The extract exhibited high antioxidant potential, reaching 185.891 ± 1.598 ppm, linked to the presence of these phytochemical compounds.

Araújo et al. (23) investigated the phenolic content of the AVDPs and AVDS cultivars of Hass and Fuerte and evaluated their potential as antioxidants, anti-inflammatory, and cytotoxic agents. AVDPs predominantly contain procyanidin, trans-5-O-caffeoyl-D-quinic acid, catechin, and epicatechin, while procyanidin B and epicatechin were predominant in AVDS, which contributes to their high radical scavenging activity. The AVDPs effectively suppress TNF-*α* and nitric oxide (NO) generation, which is associated with the high phenolic content. Therefore, the study suggests that AVDPs are a promising natural source of antioxidant and anti-inflammatory agents that could serve as a biennially useful resource in food for functional foods formulation and pharmaceutical applications.

Chlorogenic acid, also known as 5-O-caffeoylquinic acid, belongs to the hydroxycinnamic acid group and has demonstrated anti-hyperglycemic properties, superior DPPH activity compared to vitamin E, and effectiveness as an antioxidant for preventing oxidation and the formation of free radicals (64, 120, 121). Furthermore, derivatives of quercetin, representing one of the largest classes of flavanols, have been associated with controlling oxidation and inflammation, as well as serving as protective agents against cardiac diseases (93).

The antioxidant properties of AVDP extract have been widely documented by several studies. Ferreira and Santos (90) reported Hass AVDP with 93.92% DPPH inhibition, while Melgar et al. (34) reported Hass AVDP with antioxidants of EC_50_ ranging from 11.7 to 152 μg/mL (DPPH, reducing power, and *β*-carotene bleaching inhibition), which were attributed to the presence of phenolic compounds. Figueroa et al. (92) reported antioxidant activity assays using ABTS (1.34 mmol Eq T/g DE), FRAP (2.66 mmol Eq F (II)/g DE), and ORAC (3.02 mmol Eq T/g DE). It has also been reported to exhibit neuroprotective activity (122), further solidifying its potential application in the food industry. It can serve as an ingredient in many functional foods, enabling the use of AVD waste, a latent concern of the circular economy (40). Several studies also report potential use cases of the AVDP extract in addressing diseases related to oxidative stress (122–125).

#### Antimicrobial

The inefficacy of the existing and used synthetic drugs has become more pronounced and keeps increasing due to their resistance to microorganisms. In addition, the more toxic side effect associated with these synthetic drugs have limited their uses (126, 127). This resistance of pathogens to antibiotics and ineffectiveness of the antibiotics for the treatment of diseases has become alarming and has resulted from the misuse, inappropriate use, and indiscriminate use of these antibiotics. To forestall this alarming incidence of antimicrobial resistance accompanied by increasing infectious diseases, a proactive and better approaches are required (127, 128). In this respect, natural materials, including plants, plant products, and their byproduct, have been presented as an acceptable and satisfactory agent furnished with an enormous range of efficacious and active antimicrobial chemicals.

A remarkable antimicrobial potential of the synergistic effect of AVDP extract with nisin has been revealed against *Listeria innocua* (ATCC 33090), *Escherichia coli* (JMP101), *Lactobacillus sakei*, *Weissella viridescens*, and *Leuconostoc mesenteroides*. The highest 61% inhibitory activity was shown by AVDP extract against *L. innocua*, surpassing nisin’s inhibitory activity of 39% (53). Higher antimicrobial potentials of AVDP extract were reported against Gram-positive bacteria, with a strong *in vitro* antioxidant potential attributed to its richness in polyphenolic compounds compared to the pulp (54).

A strong inhibitory effect of AVDP extract against the tested bacteria was demonstrated in a study conducted by Skenderidis et al. (129). Procyanidin A and B, catechins, quercetin, glycerides, triamcinolone acetaminophen, saponins, steroids, caffeoalkinic acid, and coumaric acid, which are polyphenolic compounds, were identified as key bioactive components associated with these antimicrobial activities (34, 111, 129, 130). Based on the antimicrobial mechanisms of action of AVDP-derived bioactive compounds, the general effects included destabilization of the cytoplasmic membrane, permeabilization of the cell membrane, inhibition of extracellular microbial enzymes, disruption of microbial metabolism, and a deficiency of microbial growth substrates (primarily essential minerals), which remain the key antimicrobial mechanisms linked to proanthocyanidin type A (129, 131). The ability to disrupt cell membranes, bind with cell proteins, inhibit enzymes, deprive substrates, complex with metal ions, and interfere with essential microbial metabolic processes has been established as a major antimicrobial mechanism enacted by these bioactive compounds (129).

Effective antibacterial activity was exhibited by AVDP extract against *E. coli*, *Salmonella* spp., *Pseudomonas aeruginosa*, *Listeria monocytogenes*, *Staphylococcus aureus*, and *Bacillus cereus* at concentrations up to 750 μg/mL. However, the organic fraction showed better inhibitory effects, achieving an increase of 83.34% against *L. monocytogenes* at a MIC of 125 μg/mL, indicating an improvement of up to 25% (132). Additionally, the acid-microwave hydrolyzed APE (HAPE) demonstrated a concentration-time-dependent inhibition of biofilm formation at lower concentrations compared to amoxicillin. Ripe AVDP extract contained cardenolides, bufadienolides, 2-deoxy sugars, unsaturated steroid/triterpenoid, unsaturated lactone, and flavonoids, demonstrating a stronger inhibitory effect against *S. aureus, P. aeruginosa,* Methicillin-Resistant *S. aureus*, and five other clinical isolates (133). The studied ripe AVDP extract, a crude extract, exhibited efficient antibacterial properties when compared with the synthetic antibiotics used as the control.

The ethanolic extraction of the AVDP, as prepared and studied by Amado et al., demonstrated its antimicrobial potential was examined against *S. aureus*, *B. cereus*, *E. coli*, and *Salmonella typhi*. Effective bactericidal and bacteriostatic activities of the AVDP were revealed (113, 132). Many other studies have documented the antimicrobial efficacy of the AVDP. Ferreira and Santos (90) reported antimicrobial activities of Hass AVDP against *E. coli*, *S. aureus*, and *Staphylococcus epidermidis* with a zone of inhibition of 5.0 mm, 13.0 mm, and 14.0 mm, respectively. Melgar et al. (34) reported Hass AVDP antimicrobial activities against *B. cereus* (MIC 0.015 mg/mL, MBC 0.030 mg/mL), *L. monocytogenes*, *Micrococcus flavus*, and *S. aureus* with the same value of MIC 0.030, MBC 0.075, *Enterobacter cloacae* (MIC 0.015 mg/mL, MBC 0.030 mg/mL), *E. coli* (MIC 0.03 mg/mL, MBC 0.45 mg/mL), *P. aeruginosa* (MIC 0.030 mg/mL, MBC 0.075 mg/mL), and *Salmonella typhimurium* with MIC 0.10 mg/mL and MBC 0.15 mg/mL. Four *Aspergillus* spp., Two *Penicillium* spp. and *Trichoderma viride* were reported with the same value of MIC of 0.3 mg/mL (34). Raymond and Dykes (94), reported Hass and Fuerte AVDP antimicrobial activity ranged between 104.2–416.7 μg/mL against *L. monocytogenes, S. epidermidis*, *S. aureus*, *Enterococcus faecalis*, *E. coli*, *Salmonella Enteritidis, Citrobacter freundii, P. aeruginosa*, *S. typhimurium*, and *E. aerogenes*, and three fungi, *Aspergillus flavus*, *Penicillium* spp., and *Zygosaccharomyces bailii*. Figueroa et al. (92) reported antimicrobial activities ranged from 9.4–22.0 μg/mL against *S. epidermidis and E. faecalis*, *Enterobacter hormaechei, Kluyveromyces marxianus,* and *Galactomyces candidus.*

Amado et al. (134) compared the antioxidant properties, antibacterial properties, and toxicity effects of AVDP, AVDS, and AVDPP of Quintal, Fortuna, Margarida, and Hass varieties. The result showed the highest antioxidant and antibacterial activity against food pathogens, exhibited by Quintal variety AVDP ethanolic extract, with no toxicity in the preliminary tests, which presents AVDPs as an efficient additive, significantly useful in food formulations.

#### Anti-inflammatory

Inflammation arises in the process of the body trying to control tissue healing and eliminate foreign substances, infections, or irritants (135). Persistence in inflammatory reactions may lead to tissue deterioration, or excessive inflammatory responses may occur, which may give rise to diverse diseases in the body system; hence, management of the inflammatory reactions is necessary (136). Recently, scientists have been in search of natural materials that could be effective in reducing inflammation and alleviation pain. Inflammation was induced by carrageenan in the mice, and they were exposed to a specific concentration of infusion, decoction, and extract of AVDPs. The result revealed a significant anti-inflammatory potential of the treated with the AVDP extracts, with the best anti-inflammatory potential shown by shorter extraction time by infusion (15 min) compared to the decoction (30 min). The findings established that the extraction method and the solvent used had a great influence on the anti-inflammatory property of AVDP (135).

AVDP extract inhibits the release of the pro-inflammatory TNF-*α* and the inflammatory mediator nitric oxide. These effects may be linked to its abundance of phenolic compounds and its higher radical scavenging and antioxidant activity compared to nisin, a natural antimicrobial dipeptide (69, 137). Anti-inflammatory compounds present in AVDPs, such as trans-5-O-caffeoyl-D-quinic acid, procyanidin, and catechin, are effective anti-inflammatory agents. Procyanidins, which are found in AVDPs, represent one of the largest groups of phenolic compounds in food products and have been demonstrated by several studies to help prevent cancer, inflammation, urinary tract infections, and various chronic diseases (35, 55, 138).

Ovalle Marín et al. (139) characterized and investigated the anti-inflammatory potential of AVDP extract. Aqueous and hydroalcoholic solutions were used as a medium for extractions. The Folin-Ciocalteau technique was adopted to quantify the Total polyphenol content present. Antioxidant capacity was determined using FRAP and DPPH, while NO and TNF-*α* release, and by TNF-α gene expression were used to measure the inflammatory features of the AVD extract. The result revealed an adequate presence of polyphenol content in both. However, higher polyphenol content was reported in the hydroalcoholic extracts than in the aqueous extract. Furthermore, a pronounced and higher antioxidant activity and anti-inflammatory efficacy were revealed in the hydroalcoholic extracts than in the aqueous extract. These show that higher bioactive contents are found in hydroalcoholic extracts than in the aqueous extract, which accounts for its higher biomedical application.

Investigations into the extraction of bioactive compounds from AVDP extract were carried out by Rodríguez-Martínez et al. (11) using UAE techniques. The antioxidants and the anticancer potentials of the recovered AVDP extract were assessed against cancer cell lines. Hydroxybenzoic, hydroxycinnamic acids, flavanols, flavanonols, flavones, flavanones, chalcone, phenylethanoids, and lignans represent the most detected chemical compounds. Aqueous ethanol extracts present the highest contents of chemical composition with high antioxidant activities. The extract showed strong anticancer potentials against the Caco-2, A549, and HeLa cell lines and did not significantly affect normal cells (L929) with low cellular toxicity in normal cells used in this study. Thus, the work presented the utilization of ultrasound as a workable extraction technique and confirmed the safety of AVDP extract for human consumption. Hence, this affirms AVDP utilization as an effective agent as a functional ingredient. The therapeutic potential of the epicatechin derived from AVD has been established against diabetes and cancer (140, 141).

### Potential in the food industry

Hunger incidence is one of the global challenges, of which more than 820 million people were suffering from hunger in 2018, stressing the immense challenge and importance of achieving the Zero Hunger target by 2030 (142). In addition, antioxidant and antimicrobial agents have remained key and significant agents in the food industry. Hence, the continuous search for natural alternative food sources of high nutritional value furnished with bioactive compounds with antioxidants and antimicrobial potentials has been of great interest as an alternative to synthetic compounds, which are associated with negative health effects (143). These natural food sources will fill/cover almost 70% of the needed food materials to meet the exponentially growing human population. Bioactive compounds derived from natural materials are both safer, with no side effects on human health and the ecosystem (93, 143). Food industries are so much concerned and engrossed in the bioactive compounds with antioxidant and antibacterial potentials to impede the oxidative process and microbial contaminations in the food product, thereby improving the shelf-life and quality of products (144).

In this sense, AVDP is a rich source of different bioactive compounds such as tannins, phenolic acids, and flavonoids, including catechin and various procyanidins, flavonols, hydroxybenzoic and hydroxycinnamic acids, with antioxidant and antibacterial potentials (64, 93, 143). Extract from AVDP has been established as a good source of various phenolics, including flavanols, anthocyanins, and phenolic acids, which are known for their antioxidant abilities and use in preserving food products (52, 69). Sequel to the bioactive compounds as reported in the AVDPs, which contribute to its antioxidant and antimicrobial activities, could present AVDPs as a valuable product in the food industry. Hence, these characteristics make them a rich and natural source of bioactive compounds for applications in the food sector (143). However, it is very necessary and will be a very useful decision for the food industry to implement the circular economy to promote or prioritize the reuse or recovery of by-products [Del Rio (22, 145)].

Consumptions of AVDs are made available in different forms, including guacamole, chips, ice cream, frozen products, AVD paste, AVD oil, and cosmetic products (6, 26, 60). Applications and adaptations of the AVD as a functional component for foods has gained outstanding interest due to their bioactive compounds such as unsaturated fatty acids, phenolic compounds dietary fibre, vitamin B, C, and E, lutein, and various pigments (carotenoids, chlorophylls, and anthocyanins) (26, 55, 59, 146).

A lipid profile of the AVDP, coupled with an in-depth update on the lipid fingerprint of *Hass* seed investigated using reversed-phase liquid chromatography–tandem mass spectrometry, was investigated by Neves et al. (147). The result revealed higher lipid content in the peel than seed. The reported lipid contents showed significant antioxidant and anti-inflammatory properties. Hence, this study suggests the lipid contents from AVDP and seed are promising sources of natural and functional bioactive compounds with biotechnological importance in the food/cosmetics industry, and nutraceuticals production. In addition, AVDP and seed lipid contents serve as a source of lipids for new plant-based products development and production of nutraceuticals and cosmeceuticals in managing oxidative stress and inflammation, as well as an additive to replace synthetic antioxidants, thus enhancing the implementation of AVD peels as a sustainable raw material.

The importance of iron in the body cannot be overemphasized. Lack of iron or a low iron diet in the body leads to a disease condition known as Anemia (148). Manganese is another important element involved in various biological activities within the body system and is generally obtained from a healthy diet, as the human body does not produce manganese. Although manganese is obtained from food and stored in various organs in the body. The widespread intake of flour and flour-based products makes the conversion of AVDPs into peel flour an easy and sustainable method of improving the quality of food, and it can also serve as a vehicle for functional dietary supplements, providing the necessary macro and micro elements including iron and manganese (149). There has been an increase in demand for the utilization of AVDs in food and nutraceutical industries due to these characteristics (25).

#### Food additives and preservatives

Preservation of food and food products has gained high demand by the food industry and the consumers to enhance and sustain healthy products (138). Due to the continually increasing demand for healthy food products from consumers, the adoption and applications of natural food preservative agents have received tremendous interest (138, 150).

Microbial decomposition and sensory alterations of meat and meat products have become one of the greatest issues in the food industry and industrial production of meat (151, 152). The microbial decomposition results from the oxidation of meat components caused by the free radicals and Reactive oxygen species (ROS), greatly reducing the meat product’s shelf life, and in addition, limiting their production. The synthetic form of the antioxidant agent presently in use has been reported with several side effects (153). Hence, there is a need to search for an efficient mechanism to solve these challenges. The potential search and use of natural agents as food additives in food preservation and fortification has gained the attention of scientists in recent years as an alternative to synthetic antioxidants (34, 147, 151).

Calderón-Oliver and López-Hernández (153) demonstrated the potency of AVDP extract to prevent protein oxidation. Inhibition of lipid and protein oxidation and spoilage prevention of meat products of AVDP extract were investigated by Rodríguez-Carpena et al. (54). This present AVDP, a valuable material in the food industry as a food-grade preservative. Figueroa et al. (92) also investigated the potency of AVDP extracts in combating microorganisms, and the high antimicrobial activity displayed against both gram-positive and gram-negative bacteria further highlights the effectiveness of AVDPs as natural preservatives to extend shelf life, and prevent rapid food spoilage.

The impact of integrating the AVDP extract on the physiological and antifungal properties of the developed gelatin-carboxymethylcellulose active films containing Hass AVDP extract and their applicability in berry preservation has also been explored (187). Gelatin/carboxymethylcellulose active films were developed and incorporated with different concentrations of AVDP extract at 0, 200, 300, and 400 mg L^−1^. The best barrier properties against water vapour were recorded at the 200 mg L^−1^ concentration of AVDP extract. The result showed that incorporating AVDP extract has the efficiency to reduce the moisture content and solubility of the films and showed higher colorimetric parameters and opacity than the control film. The developed gelatin-carboxymethylcellulose radical scavenging capability (from 24.16 to 41.12, 57.21, and 63.47%) was shown to be significantly enhanced, with a robust antimicrobial inhibition against the growth of *Rhizopus stolonifer* and *Aspergillus niger* increasing with the increasing concentration of the AVDP incorporated. The preservative study was observed for 6 days of storage without any fungal development. This present AVDP extract as a natural alternative potential agent for active packaging and can preserve fresh fruit.

Velderrain-Rodríguez et al. (125) identified AVDP TPC profile and evaluated their antioxidant and antiproliferative properties. The study reported that AVDP showed the highest phenolic contents (309.95 *±* 25.33 mMol GA/100 g of extract) with the lowest effective concentration (EC50) against DPPH and ABTS radicals (72.64 *±* 10.70 and 181.68 *±* 18.47, respectively), better than the seed coat and seed extracts examined. AVDP extract antiproliferative activity was revealed, followed by the seed, then the seed coat. The meat lipids and proteins were preserved by AVDP extracts (70% acetone) by inhibiting the oxidative reactions in meat patties (54).

#### Animal feed formulation

The AVDP extract has been used in formulating animal feed due to their proximate and bioactive compound composition. These agro-based wastes could be incorporated into the animal feed or used as a raw material in the production of animal diets (19). Traditionally, AVDP has been applied and used as feed for livestock (93).

Okibe et al. (154) examined the nutritional value of the AVDPs and seeds quantitatively. Higher contents of crude fibre and protein, than that of the seeds, coupled with a non-significant difference in ash content (5.10 ± 0.00) and carbohydrates (76.21 ± 0.03), but low values in lipids and moisture content when compared with that of the seed, were reported. In addition, higher mineral content, including P (1.25 ± 0.01), Na (0.45 ± 0.00), with a non-significant difference in Ca (0.06 ± 0.00) and a lower value of Fe (0.13 ± 0.00) and Mg (0.11 ± 0.01), was revealed when compared with that of the seeds. High carbohydrate content contributes to the AVDP as a potential carbohydrate source for animal feed formulations. AVDP’s low moisture content contributes to its shelf-life extension, while its low ash contents point to a low level of inorganic impurities. Hence, present AVDP applications as an adequate source of these minerals in animal feed formulations.

#### Tea and beverages formulation

Beverages encompass drinks such as hot, soft, milk, and alcoholic varieties, generally consumed for refreshment (155). Understanding the important role of food products beyond their basic nutritional needs has led to significant advancements in the functional foods industry, and beverages serve as key vehicles for incorporating nutritional bioactive compounds into functional food products (156, 157). The ease of distribution and the advantages of beverage bioactive compounds have made them the preferred carriers for functional ingredients (156). Consequently, AVDP is seen as a promising source of functional ingredients in the beverage sector due to its richness in bioactive compounds. Dried AVDPs were used to create a novel functional beverage. Rotta et al. (68) reported that the quality of the mate tea was comparable to that of the tea produced from the AVDP, with both having similar high concentrations of phenolic compounds, which were not significantly affected during storage. This tea is rich in polyphenols and antioxidants, mirroring the characteristics of mate tea and maintaining its content throughout storage. Utilizing AVDP in beverage preparation aims to enhance their functional properties.

Rotta et al. (68) utilized AVDP for tea formulation. The AVDP tea formulation was shown to contain high phenolic and flavonoid compounds and exhibited a significant antioxidant activity. The reported phenolic and flavonoid contents in this study were found to be higher than apple and mate tea. The tea prepared using AVDP displayed a suitable sensory analysis, while containing a high content of phenol, was seen to be present and exhibiting significant antioxidant activity.

#### Juice clarification

The application and importance of pectinolytic enzymes have received global attention from various scientists in catalyzing a diverse range of industrial processes. Notably, microorganisms have been used in the production of various types of pectinolytic enzymes and have been reported to account for about 25% of food enzymes sales worldwide, with steadily increasing market shares (158, 159). Pectinolytic enzymes are used in various industrial applications (160–166). However, some agricultural waste, like AVDP, has been established as an alternative source of bacterial pectinase.

Pectinase-producing bacteria are mostly used by many sectors, most especially in the food industry. Significantly, pectinase is used to break down pectin polysaccharide compounds. Haile et al. (167) isolated four different pectinase-producing bacteria strains, including *Serratia marcescens* and *Lysinibacillus macrolides* from AVDP, while their potential in making juice clarification was evaluated. Clear apple, lemon, and mango juices were achieved and further processed to analyze the properties of each juice. Lemon juice presented the highest content of total titratable acidity, total phenols, and the highest antioxidant activities, while the apple juice was presented with the highest total soluble solids, reducing sugar content, and viscosity, and the mango juices showed the highest pH values. This presents AVDP as an alternative rich source of bacterial pectinase to microorganisms that could clear fruit juices.

### Biotechnological applications of avocado peels

The applications of adsorbent agents for the removal of contaminants have garnered special attention recently due to their simplicity in design and ability to produce high-quality effluents (60, 168). Carbonaceous material produced from the AVDP was used for dye removal in place of the conventional activated carbons, which are restricted in use due to their high costs and are limited by their exhaustion after long-term operations. Efficacy and possibility of the produced carbonaceous material were evidenced by the removal of various dyes. The result showed a complete removal of Naphthol Blue Black, Reactive Black 5, and Blue 41. Hence, the study presents the effective dye removal of the carbonaceous material produced from the AVDP as a low-cost, easily accessible, proven alternative to conventional and synthetic adsorbents.

The search for naturally activated carbon as an effective alternative to the commercial activated carbons (CAC) to reduce or remove the chemical and biological oxygen demand in processing wastewater has recently increased (169). The existing use of the commercial activated carbons (CAC) from peat, coal, or petroleum pitch is effective as adsorbents but very expensive, which has resulted in the search for alternative materials to CAC (169, 170). Sequel to this, agricultural wastes have been an alternative source for the production of activated carbon in recent times and are therefore considered as the most accessible and cheap carbonaceous materials instead of CAC (169, 171). Different agricultural wastes, husk, wood, palm kernel, and AVD seeds have been employed in the assessment of bioremediation ability, and AVD by-products have been verified for their effectiveness as adsorbents in bioremediation (169–171).

AVD has been revealed as a potential agent for the production of activated carbon. Employing AVDP as a source of activated carbon serves as a cheaper substitute compared to existing high-priced activated carbon. It was shown that the adsorption capacity of AVDP activated Carbon was equivalent to the commercial produced ones. However, the quality of AVDP-treated water was reported to be more suitable for irrigation and safer for direct discharge to the water sources (35, 172). Applications of AVDPs as a precursor for AC synthesis can potentially solve the disposal problem and add value to the agricultural residue (173). The high percentage of starch contained in the AVDPs serves as a good indicator of their high carbon content in comparison with other agricultural wastes, resulting in high AC yield (174). Hence, the utilization of AVDPs as a biosorbent can solve the problem of managing the large amount of AVD waste.

AVDP produces ecology-friendly adsorbents that could be used for the removal of acidic and alkaline dyes instead of conventional activated carbons. Palma et al. (60) conducted a study, optimizing the conditions for the process, applying factorial design and response surface methodology at a carbonization temperature of 900 °C for 65 min. Carbonized AVDP is a promising adsorbent for removing different types of dye due to the wide availability of AVDP and its subsequent low cost, coupled with its potent adsorption capacity.

An innovative biomass solid catalyst recovered from AVDP was developed by Etim et al. (175), and its potential in the transesterification of a bi-hybrid oil of used cooking–baobab oil (UC-BO) was evaluated. The result showed AVDPs rich in vital elements with high concentrations of K content. An effective catalytic potential of the AVDP catalyst was also revealed by converting the used cooking–baobab oil to biodiesel. The AVDP catalyst displayed an exceptional recyclability, attaining 92.85% biodiesel yield. The study considered a developed catalyst as a highly effective biomass-based catalyst for large-scale biodiesel production. Epicatechin and chlorogenic acid have also been reported to inhibit the formation of heterocyclic amines in charcoal-roasted lamb meats (176). AVDP extract, therefore, presents itself as a product with immense biotechnological potential, with applications in the production of colorants, biopolymers, natural antioxidants, and more.

### Cosmetics/skin care effect of avocado peels

The search for a natural source of cosmetic ingredients by the cosmetic industry in the replacement of synthetic substances has currently received high demand (93). This is due to several side effects of the synthetic substances, including carcinogenic effects and many more. There has been a consistently increasing demand for the utilization of natural raw materials to replace synthetic substances (177). Agricultural by-products, therefore, pose as a promising alternative in providing these natural ingredients (55). AVDP has shown promise in cosmetic product incorporation, such as oil-in-water emulsions. Their incorporation enhanced the antioxidant and antibacterial potency of the cosmetic products. This shows the AVDP as a promising alternative to the synthetic additives applied in the preparation and manufacture of cosmetics.

Ferreira et al. (90) obtained AVDP extract and incorporated it in oil-in-water and water-in-oil types of cosmetic formulations, and compared their stability with formulations containing synthetic preservatives. Based on the stability evaluation, extract from AVDP showed efficient use in the studied emulsion and specifically enhanced the antioxidant and antibacterial properties of the formulated emulsion. This further proves AVDP as a viable option to replace synthetic preservatives, proving more effective and stable. This suggests the feasibility of obtaining sustainable cosmetics by incorporating AVDP extracts, which serve as a low-cost, easily accessible, and eco-friendly alternative source of phenolic compounds.

## Conclusion

AVDP serves as a rich source of diverse bioactive compounds that remain key components to several industries, most especially the food, pharmaceutical, and cosmeceutical industries, which, in addition, promote the circular economy agenda for zero waste and increase the economic status of the countries. They are rich in nutritional composition, which promotes the significant use in the food formulation/preservation and contains vital bioactive compounds including phenolic and flavanols like epicatechin, procyanidin, quercetin, chlorogenic acid, chlorophyll a and b, and many more. AVDP had diverse biological activities, like antimicrobial, antioxidant, and anti-inflammatory activities. Generally, different factors like the morphological appearance ripe and unripe, geographical origin, growth conditions, extraction methods, extraction solvent, temperature range, and others had a great effect on the quality, quantity, and the biological activities of the AVDP bioactive compounds. Hence, AVDPs serve as a promising agent in different industries, including food as functional food and food preservatives, in pharmaceuticals and cosmeceuticals containing vital bioactive compounds with great biomedical applications for health improvement and better economic performance.
